# miR-146b-5p serves as a novel biomarker for acute myocardial infarction and is involved in regulating oxidized low-density lipoprotein-induced endothelial injury through targeting PRKAR2A

**DOI:** 10.1186/s41065-026-00706-6

**Published:** 2026-07-09

**Authors:** Hailiang Wu, Hui Zhang, Yuyan Hou, Zhifang Wang, Yang Bian

**Affiliations:** 1 Department of Cardiology, Cardio-Cerebral Vascular Diseases Hospital of General Hospital of Ningxia Medical University, Yinchuan City, Ningxia China; 2 Department of Cardiovascular Medicine III, Xinxiang Central Hospital, Xinxiang City, Henan Province China; 3 Department of Cardiology, Baoji Hospital of Traditional Chinese Medicine, No.58, Xingzheng Avenue, Jintai District, Baoji City, Shaanxi Province 721004 China

**Keywords:** miR-146b-5p, PRKAR2A, Acute myocardial infarction, Endothelial cell injury

## Abstract

**Background:**

MiR-146b-5p, a disease-responsive miRNA with circulatory stability, has been implicated in multiple pathologies, but its role in acute myocardial infarction (AMI) remains unexplored. The objective of this study was to explore the role of miR-146b-5p in AMI.

**Methods:**

Serum from 103 AMI patients and 95 healthy controls was analyzed via qRT-PCR to quantify miR-146b-5p/PRKAR2A expression, with ROC curves evaluating diagnostic performance and correlation analyses linking miR-146b-5p levels to clinical indicators of AMI severity. Prognostic associations were systematically assessed using Kaplan-Meier curves and COX regression models. In vitro ox-LDL-induced HCAEC injury models employed CCK-8 assays, flow cytometry, Caspase-3 activity measurements, ELISA, and oxidative stress assays to elucidate cellular mechanisms. Dual-luciferase reporter and Western blot assays assessed the relation between miR-146b-5p and PRKAR2A.

**Results:**

Upregulated miR-146b-5p expression was observed in AMI compared with the HC group, showing diagnostic value and correlation with hs-CRP, CK-MB, cTnI, LVEF, and Killip grade. Higher miR-146b-5p expression predicted poor prognosis of AMI. In ox-LDL-induced HCAECs, miR-146b-5p inhibition reduced apoptosis, inflammation (IL-1β, TNF-α), and endothelial dysfunction marker levels (ET-1, vWF), while promoting proliferation. PRKAR2A was downregulated in ox-LDL and confirmed as a miR-146b-5p target, with its knockdown reversing the protective effects of miR-146b-5p inhibition on ox-LDL-induced HCAEC injury.

**Conclusions:**

Upregulated miR-146b-5p, which correlated with AMI severity, demonstrated diagnostic and prognostic potential for AMI. It promoted AMI progression by aggravating endothelial injury under ox-LDL induction through suppressing PRKAR2A.

**Supplementary Information:**

The online version contains supplementary material available at https://doi.org/10.1186/s41065-026-00706-6.

## Background

Coronary atherosclerosis, marked by the gradual buildup of lipid-laden plaques in the arterial intima, serves as the fundamental pathological basis for acute myocardial infarction (AMI) [1]. This life-threatening event is triggered by plaque rupture, ensuing thrombus formation, and complete arterial blockage, ultimately leading to irreversible myocardial ischemia and necrosis [2]. As the most lethal manifestation of coronary artery disease (CAD), AMI accounts for approximately 10 million annual cases worldwide and causes a substantial public health burden [3]. Contemporary diagnostic approaches of AMI typically require more than 50% coronary stenosis on angiography and objective ischemia evidence, including electrocardiography (ECG) changes and elevated cardiac troponin [4]. However, these methods face notable limitations, including the atypical symptoms in elderly or diabetic patients, cardiac troponin can rise in non-cardiac conditions (such as kidney disease), early AMI may show subtle ECG changes, and angiography is invasive and not always available emergently [5, 6]. While percutaneous coronary intervention (PCI) and thrombolysis enable timely reperfusion (< 12-hour window), their clinical efficacy is constrained by the no-reflow phenomenon due to microvascular obstruction, and reperfusion-induced arrhythmias [7, 8]. Pharmacological approaches combining dual antiplatelet therapy with high-intensity statins demonstrate robust secondary prevention benefits, yet these regimens remain fundamentally limited in their inability to modify plaque vulnerability or halt disease progression [9]. The diagnostic and therapeutic limitations highlighted above underscore the urgent need for novel biomarkers to enable widespread screening and precise interventions in AMI clinical management.

It is established that vascular endothelial injury serves as the central initiating factor in the occurrence and progression of AMI, permeating the entire continuum from atherosclerosis formation and unstable plaque rupture to thrombus formation. MicroRNAs (miRNAs), a class of non-coding RNAs, regulate gene expression post-transcriptionally through sequence-specific binding to target mRNAs, thereby playing critical roles in regulating cellular processes [10]. Notably, the involvement of miRNAs in the AMI process, including the regulation of vascular endothelial cell function, has been extensively investigated. For instance, upregulated miR-19a and miR-499 demonstrate diagnostic and prognostic potential for AMI [11, 12]. Furthermore, miR-32-5p, which is identified as a diagnostic biomarker for AMI, has been shown to promote endothelial injury by reducing endothelial cell viability, highlighting its therapeutic implications through the modulation of endothelial function [13]. The research on dysregulated miRNAs in AMI progression underscores their significant potential as novel biomarkers for AMI clinical management.

Emerging evidence from meta-analyses and clinical studies has consistently demonstrated that miR-146b-5p was upregulated in both CAD and atherosclerosis [14, 15]. In addition, upregulation of miR-146b-5p is also observed in TNF-α-induced endothelial cell inflammation [16]. Given the close association among endothelial cell injury, atherosclerosis, and AMI [17, 18], it could be speculated that miR-146b-5p may participate in AMI pathogenesis. However, its specific expression and mechanistic involvement in AMI remain unexplored. Based on the above findings, it could be hypothesized that miR-146b-5p was dysregulated in AMI and might be involved in the regulation of AMI progression. This study aimed to investigate the expression profile of miR-146b-5p in AMI patients and evaluate its potential as a novel biomarker for AMI diagnosis, and provide novel insights for AMI clinical management.

## Subjects, materials, and methods

The study workflow could be observed in the Supplementary Fig. 1.

### Subjects

The study population comprised 103 patients initially diagnosed with AMI (AMI group, *n* = 103) through ECG and coronary angiographic confirmation at Xinxiang Central Hospital between 2023 and 2024, and 95 healthy individuals (healthy control group, HC group, *n* = 95) who underwent routine physical examinations during the same period. The sample size was validated using G*Power post hoc analysis (effect size: 0.3, α err probability: 0.05), which achieved a power of 0.99. All procedures were conducted in accordance with the ethical guidelines established by the Ethics Committee of Xinxiang Central Hospital and the Helsinki Declaration, with written informed consent obtained from all participants or their legal guardians after a comprehensive explanation of the study objectives.

#### ‌Inclusion criteria for AMI patients

(1) the age should be over 18 and less than 80 years old; (2) according to the 2017 ESC guidelines for the management of AMI, the diagnosis requires meeting at least two of the following typical criteria [19]: acute chest pain persisting for > 20 min, new-onset ST-segment elevation in two contiguous leads or ST depression in precordial leads, and significant elevation of cTnI; (3) comprehensive clinical documentation available for analysis.

#### ‌Exclusion criteria for AMI patients

(1) concurrent severe cardiovascular or cerebrovascular conditions including but not limited to: stroke, malignant hypertension, heart failure, or life-threatening arrhythmias; (2) significant impairment of major organ systems; (3) active severe infections including malignancy, sepsis, or septicemia; (4) current use of disease-modifying medications or broad-spectrum antibiotics; (5) history of major cardiovascular interventions within 90 days (PCI operation, coronary artery bypass grafting); (6) pregnancy or lactation.

The study enrolled healthy subjects aged 18–80 years with complete clinical data. Exclusion criteria included: (1) history of coronary artery disease, stroke, or peripheral artery disease; (2) chronic inflammatory/autoimmune disorders or malignancies; (3) severe organ dysfunction; (4) use of antiplatelet agents, anticoagulants, or statins within 3 months; (5) pregnancy or lactation; (6) refusal to provide informed consent; and (7) acute infections or recent surgery (< 6 weeks).

### Cells

Human coronary artery endothelial cells (HCAECs) were purchased from American Type Culture Collection (USA).

### Methods

#### Clinical trials

The study systematically collected comprehensive clinical data from participants, including demographic characteristics (age and gender), anthropometric measurements on body mass index (BMI), medical history (hypertension, hyperglycemia, hyperlipidemia), and lifestyle factors (smoking status). Fasting venous blood samples were obtained from all subjects within 24 h of hospitalization for biochemical analysis. Lipid profiles including total cholesterol (TC), triglycerides (TG), high-density lipoprotein cholesterol (HDL-C), low-density lipoprotein cholesterol (LDL-C); cardiac biomarkers including cardiac troponin I (cTnI), creatine kinase-MB (CK-MB); inflammatory markers such as high-sensitivity C-reactive protein (hs-CRP) were measured. The severity of AMI was evaluated according to the Killip classification [20]. Following centrifugation at 1500 g for 10 min, serum was separated and stored at -80 °C for subsequent analysis.

Follow-up study: All AMI subjects were prospectively followed for 6 months to assess progression-free survival (PFS) rate [21, 22]. PFS was defined as the absence of AMI recurrence and major adverse cardiovascular events (MACE) encompassing cardiovascular death, recurrent myocardial infarction, stroke, and repeat revascularization.

#### Cell culture, stimulation, and transfection

HCAECs were cultured in the DMEM culture medium supplied with 10% fetal bovine serum (FBS, Gibco, USA) and 1% antibiotics at 37 °C in a humidified 5% CO₂ incubator. Cells were passaged at 80–90% confluence using 0.25% trypsin-EDTA, and experiments were conducted between passages 3–6.

For oxidized low-density lipoprotein (ox-LDL) treatment, cells were incubated with ox-LDL at a concentration of 50 µg/mL for 24 h [23].

Transfection of ox-LDL-induced HCAECs was conducted by using Lipofectamine 3000 (Invitrogen). For miR-146b-5p modulation, cells received miR-146b-5p inhibitor (Thermo Fisher Scientific, USA) for knockdown or miR-146b-5p mimic (Thermo Fisher Scientific, USA) for overexpression. PRKAR2A silencing was achieved using small interfering RNA (si PRKAR2A, GenePharma, China). Corresponding negative controls of the inhibitor, mimic, and small interfering RNA were transfected in parallel. Cells were harvested for subsequent experiment after 48 h of incubation post-transfection.

#### Quantitative Real-Time Polymerase Chain Reaction (qRT-PCR)

Total RNA was isolated from serum and cell samples using TRIzol reagent (Invitrogen, USA) following the manufacturer’s protocol. RNA quality was assessed spectrophotometrically. The TaqMan miRNA RT Kit (Thermo Fisher Scientific, USA) was used to synthesize the cDNA. A 7500 Fast Real-Time PCR System (Applied Biosystems, USA) was employed to perform qRT-PCR using SYBR Green Master Mix (Thermo Fisher Scientific, USA) for both miR-146b-5p and PRKAR2A expression. The running condition was: 95 °C for 2 min, followed by 40 cycles of 95 °C for 3 s and 60 °C for 30 s. Data were normalized to cel-miR-39 (for miR-146b-5p) and GAPDH (for PRKAR2A) using the 2^−ΔΔCt^ method. The primer sequences were listed as: miR-146b-5p forward 5’-ATAGGATCCAGACCCTCCCTGGAATAGGA-3’, reverse 5’-ATAGGATCCGAGCACTGAGGAAGGACCAG-3’; cel-miR-39 forward 5’-AGCCCGTCACCTGGTGTAAATC-3’, reverse 5’-CAGTGCAGGGTCCGAGGTAT-3’; PRKAR2A forward 5’-CTTATTGACTAGTGCCACCATGGGTAGCCACATCCAGATCCCG-3’, reverse 5’-CCCAAGACAATTGCTACTGCCCGAGGTTGCC-3’; GAPDH forward 5’-CACCCACTCCTCCACCTTTG-3’, reverse 5’-CCACCACCCTG TTGCTGTAG-3’.

#### Cell proliferation

The cell counting kit-8 (CCK-8, Dojindo, Japan) assessed the HCAEC proliferation. Cells (1 × 10^4^ cells/well) in the 96-well plates were cultured for different time periods (0, 24, 48, 72 h). Following treatment, 10 µL CCK-8 reagent was added to each well, and plates were incubated for 2 h at standard culture conditions. Absorbance at 450 nm was measured using a microplate reader (BioTek, USA).

#### Cell apoptosis

The FITC Annexin V Apoptosis Detection Kit (BD Biosciences, USA) was used to evaluate the HCAEC apoptosis. Briefly, cells were harvested with a non-enzymatic dissociation solution (Gibco, USA), washed twice with cold PBS, and resuspended in 100 µL binding buffer. After adding 5 µL Annexin V-FITC and 5 µL PI, samples were incubated for 15 min at room temperature in the dark, and then analyzed by flow cytometry (BD FACSVerse, BD Biosciences, USA).

Caspase-3 activity was quantified using a colorimetric assay (Beyotime, China) with the specific substrate Ac-DEVD-pNA. Following a 2-hour incubation at 37 °C, the reaction was terminated, and the absorbance was measured spectrophotometrically at 405 nm.

#### Cell inflammation

Commercially available enzyme-linked immunosorbent assay (ELISA) kits (Shanghai mlbio, China) assessed the inflammatory cytokines (interleukin-1β: IL-1β, tumor necrosis factor-α: TNF-α) in HCAEC supernatants. Briefly, cell culture supernatants were collected and centrifuged at 1000 × g for 20 min to remove debris. Standards and samples (100 µL/well) were added to pre-coated 96-well plates and incubated for 1.5 h at 37 ℃. After washing, biotinylated detection antibodies were added (1-hour incubation), followed by HRP-conjugated streptavidin (30-minute incubation). Color development was achieved using TMB substrate (15-minute incubation), stopped with 2 N H_2_SO_4_, and absorbance read at 450 nm using a microplate reader (BioTek, USA).

#### Cell oxidative stress

Malonaldehyde (MDA) and superoxide dismutase (SOD) levels in HCAEC lysates were measured using commercial assay kits (Beyotime, China). For MDA quantification, samples were mixed with thibabituric acid reagent, heated at 100 °C for 15 min, and centrifuged at 1000 × g for 10 min at 4 ℃. Supernatant absorbance was read at 532 nm. SOD activity was determined using the WST-8 method, where samples were incubated with the reaction mixture for 30 min at 37 °C, and the absorbance was measured at 450 nm.

#### Endothelial dysfunction markers

Endothelin-1 (ET-1) and von Willebrand factor (vWF) levels in HCAEC supernatants were quantified using commercial ELISA kits (Shanghai mlbio, China). Cell supernatants were incubated in pre-coated plates for 30 min, followed by sequential incubations with detection antibodies and HRP-streptavidin. Color development was conducted by adding the TMB substrate and incubating for 15 min. The reaction was terminated with the stopping solution, and absorbance at 450 nm was immediately measured using a BioTek plate reader.

#### Bioinformatics analysis

Potential downstream targets of miR-146b-5p were predicted using Starbase (https://rnasysu.com/encori/index.php) and miRDB (https://mirdb.org/) databases. The overlapped targets from both databases were visualized using a Venn Diagram. DAVID Functional Annotation tools (https://david.ncifcrf.gov/tools.jsp) were used to evaluate the Gene Ontology (GO) enrichment analysis, including biological process (BP), molecular function (MF), cellular component (CC), and Kyoto Encyclopedia of Genes and Genomes (KEGG) pathway analysis.

#### Dual-luciferase reporter assay

The PRKAR2A mRNA 3’UTR fragment containing predicted miR-146b-5p binding sites (identified via miRDB) was synthesized and inserted into the pGL3 vector to generate both wild-type (PRKAR2A-WT) and mutant (PRKAR2A-MUT, with binding site mutations) constructs. These vectors were individually co-transfected with miR-146b-5p mimic/inhibitor or their corresponding negative controls (mimic NC/inhibitor NC) into HCAECs using Lipofectamine 3000. Following 48-hour incubation, the Promega Dual-Glo Luciferase Assay System quantified the luciferase activities.

#### Western blot analysis

To assess PPKAR2A protein expression, total protein in the HCAECs was extracted using radioimmunoprecipitation assay (RIPA) lysis buffer (Sangon Biotech, China) and quantified via a BCA assay. Equal amounts of protein were separated by SDS-PAGE and transferred onto a polyvinylidene difluoride membrane. The membrane was blocked, then incubated overnight with a primary antibody specific to PPKAR2A (1:2000, Proteintech, USA) and GAPDH (1:2000, Proteintech, USA), followed by a corresponding HRP-conjugated secondary antibody. Protein bands were visualized using enhanced chemiluminescence (ECL). The levels of PPKAR2A were normalized to GAPDH, which was used as a loading control.

### Statistical analysis

SPSS (v26.0) and GraphPad Prism (v9.0) were employed to perform the statistical analyses. Continuous data were expressed as mean ± standard deviation (SD) with normal distribution assessed by the P-P plot. Two-group comparisons used t-tests, while one-way ANOVA with Tukey’s post hoc test assessed multiple groups. Categorical variables were analyzed with chi-square tests. Pearson correlation assessed relationships between continuous variables. The receiver operating characteristic (ROC) curve analysis evaluated the diagnostic value of miR-146b-5p. Survival analysis was conducted by using the Kaplan-Meier (K-M) curves with log-rank tests, and Cox proportional hazards regression models were constructed to identify independent prognostic factors. All in vitro cell experiments were performed with at least three biological replicates, and each biological replicate contained three technical replicates. A *p*-value < 0.05 was considered statistically significant for all analyses.

## Results

### The clinical information of the subjects

The demographic and clinical parameters of study participants are systematically summarized in Table 1. The AMI cohort (*n* = 103) comprised 62 males and 41 females with a mean age of 55.47 ± 9.85 years and a BMI of 22.64 ± 1.48 kg/m², while the HC group (*n* = 95) included 58 males and 37 females with comparable baseline characteristics (53.80 ± 9.54 years, 22.54 ± 1.19 kg/m²). Comprehensive statistical analysis confirmed no group differences in key covariates, including age (*p* = 0.229), gender distribution (*p* = 0.902), BMI (*p* = 0.638), or prevalence of hypertension (*p* = 0.131), hyperglycemia (*p* = 0.088), hyperlipidemia (*p* = 0.152), and smoking status (*p* = 0.812). However, the AMI group demonstrated markedly elevated TC (*p* = 0.002), LDL-C (*p* = 0.042), CK-MB (*p* < 0.001), hs-CRP (*p* < 0.001), and cTnI (*p* < 0.001), accompanied by reduced HDL-C (*p* < 0.001) and LVEF (*p* < 0.001) compared to controls.

**Table 1:** The baseline information of the subjects

Parameters	HC (*n* = 95)	AMI (*n* = 103)	*p* value
Age (years old)	53.80 ± 9.54	55.47 ± 9.85	0.229
Gender (male, *n*%)	58 (61.05%)	62 (60.19%)	0.902
BMI (kg/m^2^)	22.54 ± 1.19	22.64 ± 1.48	0.638
Hypertension (*n*%)	23 (24.21%)	35 (33.98%)	0.131
Hyperglycemia (*n*%)	17 (17.89%)	29 (28.16%)	0.088
Hyperlipidemia (*n*%)	26 (27.27%)	38 (36.89%)	0.152
Smoke (*n*%)	39 (41.05%)	44 (42.72%)	0.812
TC (mmol/L)	3.75 ± 0.45	3.96 ± 0.50	0.002
TG (mmol/L)	1.61 ± 0.19	1.65 ± 0.22	0.148
HDL-C (mmol/L)	1.48 ± 0.19	1.33 ± 0.15	< 0.001
LDL-C (mmol/L)	2.75 ± 0.33	2.87 ± 0.42	0.042
cTnI (ng/mL)	-	1.78 ± 0.55	-
CK-MB (U/L)	13.07 ± 2.60	71.25 ± 10.53	< 0.001
hs-CRP (mg/L)	2.80 ± 0.48	10.14 ± 1.36	< 0.001
LVEF (%)	59.11 ± 4.57	47.12 ± 5.77	< 0.001

*HC *healthy control, *AMI *acute myocardial infarction, *BMI *body mass index, *TC *total cholesterol, *TG *triglyceride, *HDL-C *high-density lipoprotein cholesterol, *LDL-C *low-density lipoprotein cholesterol, *cTnI *cardiac troponin I, *CK-MB *creatine kinase isoenzyme-MB, *hs-CRP *high-sensitivity C-reactive protein, *LVEF *left ventricular ejection fraction

### The miR-146b-5p expression and its association with AMI

**Fig. 1:**
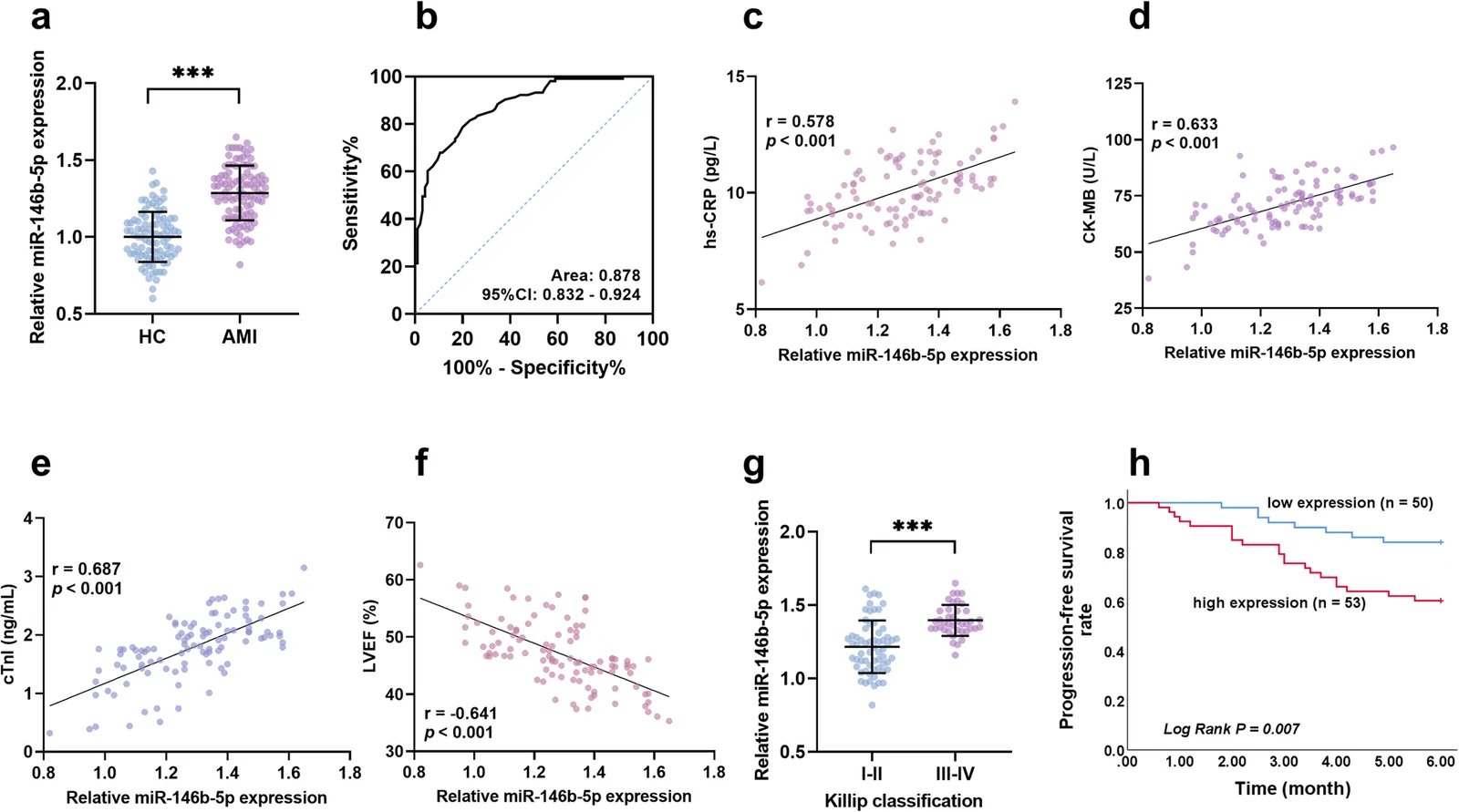
The miR-146b-5p expression and its association with AMI. **a** Upregulated miR-146b-5p expression in AMI subjects; (**b**) ROC curve identified miR-146b-5p diagnostic potential in AMI; (**c**-**f**) Upregulation of miR-146b-5p was correlated with hs-CRP(**c**), CK-MB (**d**), cTnI (**e**), and LVEF (**f**); (**g**) AMI subjects with advanced Killip grade (III-IV) showed higher miR-146b-5p expression; (**h**) K-M survival analysis indicated higher miR-146b-5p expression was associated with poor prognosis in AMI subjects. ***p < 0.001

Significantly upregulated miR-146b-5p expression was observed in AMI subjects (*p* < 0.001) compared to healthy controls (Fig. 1a), demonstrating robust diagnostic performance with an AUC of 0.878 (cut-off: 1.125, 95% CI: 0.832–0.924), sensitivity of 78.64%, and specificity of 80.00% (Fig. 1b). Upregulated miR-146b-5p levels exhibited strong positive correlations with myocardial injury biomarkers including hs-CRP (*r* = 0.578, *p* < 0.001, Fig. 1c), CK-MB (*r* = 0.633, *p* < 0.001, Fig. 1d), and cTnI (*r* = 0.687, *p* < 0.001, Fig. 1e), while inversely correlating with cardiac function as evidenced by its negative association with LVEF (*r* = -0.641, *p* < 0.001, Fig. 1f). Additionally, miR-146b-5p showed progressive elevation with disease severity, as AMI subjects with Killip grade III-IV exhibited significantly higher miR-146b-5p expression than AMI subjects with grade I-II (*p* < 0.001, Fig. 1g).

AMI subjects were stratified by average miR-146b-5p expression into a high miR-146b-5p expression group (*n* = 53) and a low miR-146b-5p expression group (*n* = 50). K-M survival analysis (Fig. 1h) revealed a worse PFS rate in the high-expression cohort (log-rank *p* = 0.007) in comparison to that of the low-expression group. Multivariate Cox regression (Table 2), after adjustment for potential confounding factors including age, sex, BMI, hypertension, hyperglycemia, hyperlipidemia, and smoking status, identified miR-146b-5p upregulation as the strongest predictor of poor prognosis (HR = 7.299, 95% CI: 1.919–27.758, *p* = 0.004), surpassing below indicators including elevated cTnI (HR = 4.729, 95% CI: 1.083–20.656, *p* = 0.039), CK-MB (HR = 4.267, 95% CI: 1.014–17.958, *p* = 0.048), reduced LVEF (HR = 0.176, 95% CI: 0.033–0.926, *p* = 0.040), and advanced Killip grade (HR = 5.394, 95% CI: 1.299–22.392, *p* = 0.020).

**Table 2:** COX regression analysis of independent factors for the AMI poor prognosis

Parameters	HR	95.0% CI		*p* value
		Lower limit	Upper limit	
miR-146b-5p	7.299	1.919	27.758	0.004
Age	1.597	0.604	4.224	0.345
Gender	2.269	0.827	6.223	0.111
Hypertension	1.240	0.523	2.940	0.625
Hyperglycemia	1.511	0.638	3.575	0.348
Hyperlipidemia	2.323	0.699	7.721	0.169
Smoke	1.139	0.431	3.004	0.793
BMI	1.027	0.377	2.792	0.959
TC	1.860	0.629	5.499	0.262
TG	1.478	0.586	3.728	0.408
HDL-C	0.591	0.229	1.524	0.276
LDL-C	1.083	0.398	2.948	0.876
cTnI	4.729	1.083	20.656	0.039
CK-MB	4.267	1.014	17.958	0.048
hs-CRP	2.487	0.750	8.248	0.136
LVEF	0.176	0.033	0.926	0.040
Killip grade	5.394	1.299	22.392	0.020

*AMI *acute myocardial infarction, *HR *hazard ratio, *CI *confidence interval, *BMI *body mass index, *TC *total cholesterol, *TG *triglyceride, *HDL-C *high density lipoprotein cholesterol, *LDL-C *low density lipoprotein cholesterol, *cTnI *cardiac troponin I, *CK-MB *creatine kinase isoenzyme-MB, *hs-CRP *high-sensitivity C-reactive protein, *LVEF *left ventricular ejection fraction

### MiR-146b-5p knockdown attenuated ox-LDL-induced HCAEC injury

**Fig. 2:**
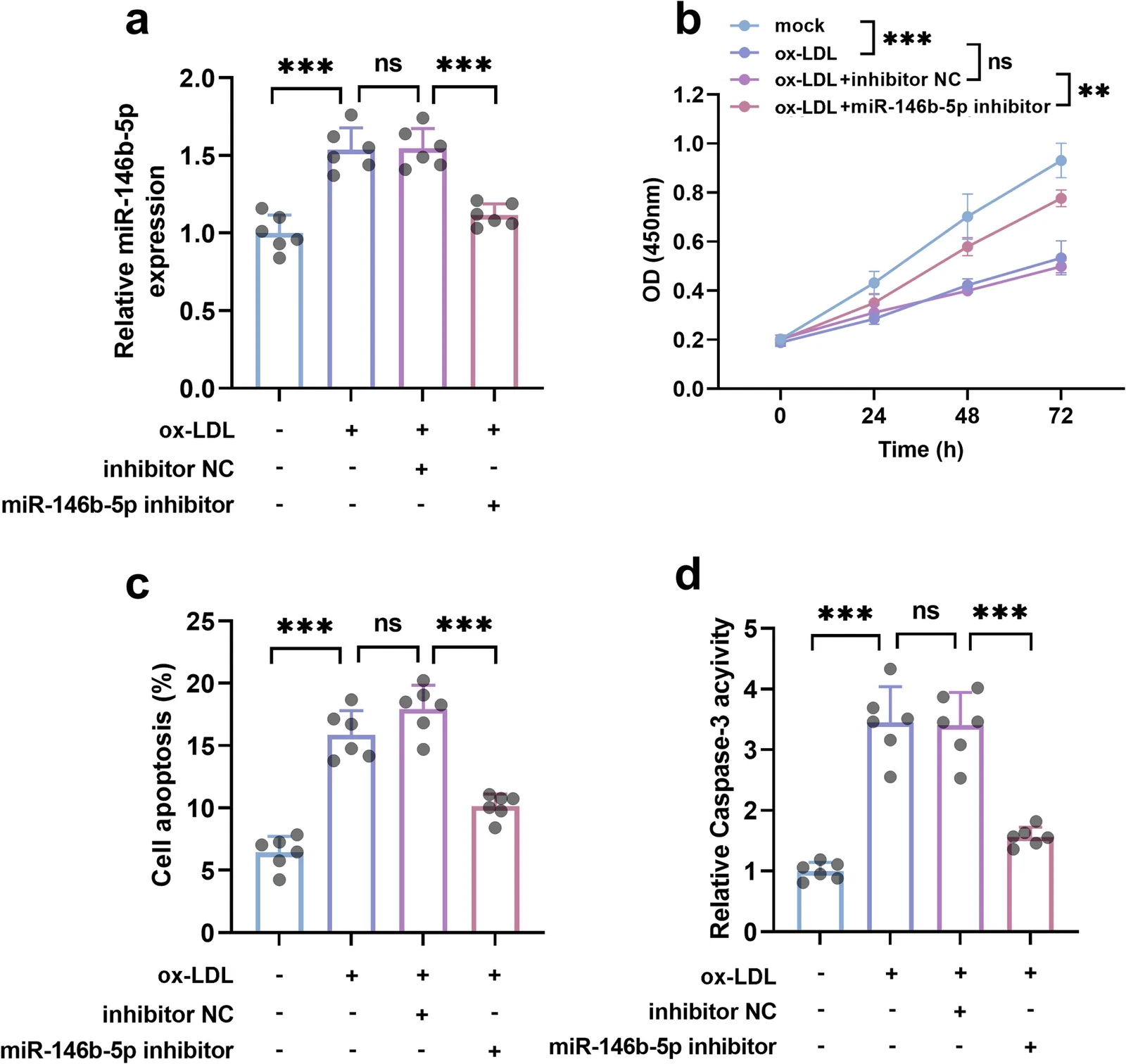
The miR-146b-5p expression in ox-LDL-induced HCAECs and its effect on cell proliferation and apoptosis. **a** Upregulated miR-146b-5p expression in ox-LDL-induced HCAECs was suppressed by miR-146b-5p knockdown; (**b**-**d**) MiR-146b-5p knockdown promoted HCAEC proliferation (**b**), decreased HCAEC apoptosis (**c**), and suppressed the caspase-3 activity (**d**) in ox-LDL-induced HCAECs. ns: no significance, **p < 0.01, ***p < 0.001

Under ox-LDL induction, HCAECs exhibited significant upregulation of miR-146b-5p expression (*p* < 0.001, Fig. 2a), which was effectively attenuated through targeted miR-146b-5p knockdown (*p* < 0.001). The stimulation of ox-LDL triggered characteristic cellular responses including impaired proliferative capacity (*p* < 0.001, Fig. 2b), enhanced apoptotic rates (*p* < 0.001, Fig. 2c), and upregulated caspase-3 activity (*p* < 0.001, Fig. 2d) Notably, miR-146b-5p inhibition demonstrated protective potential by partially rescuing these ox-LDL-induced alterations through restoring proliferation ability (*p* = 0.001) while mitigating apoptotic cell death (*p* < 0.001).

**Fig. 3:**
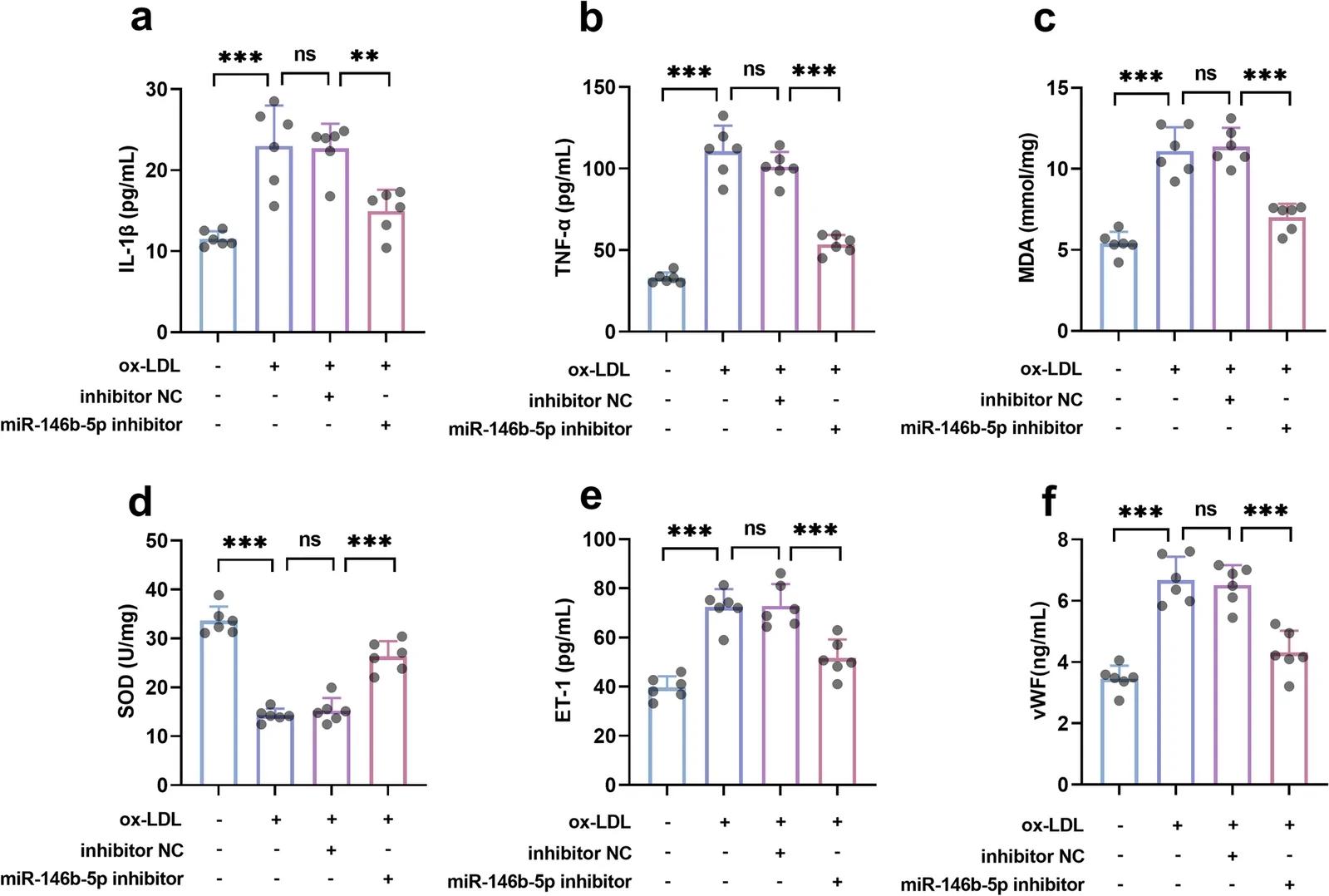
The effect of miR-146b-5p on ox-LDL-induced HCAEC inflammation, oxidative stress, and levels of endothelial dysfunction markers. **a**-**b** miR-146b-5p knockdown attenuated ox-LDL-induced increases in IL-1β (**a**) and TNF-α (**b**) levels in HCAECs; (**c**-**d**) Knockdown of miR-146b-5p reduced MDA production (**c**) and enhanced SOD activity (**d**) in ox-LDL-induced HCAECs; (**e**-**f**) MiR-146b-5p knockdown decreased the expression of ET-1 (**e**) and vWF (**f**) in ox-LDL-induced HCAECs. ns: no significance, **p < 0.01, ***p < 0.001

Ox-LDL-induced endothelial dysfunction in HCAECs was characterized by a pro-inflammatory cytokine surge, including elevated IL-1β (*p* < 0.001, Fig. 3a) and TNF-α (*p* < 0.001, Fig. 3b) levels, oxidative stress exacerbation as demonstrated by increased MDA (*p* < 0.001, Fig. 3c) and reduced activity of SOD (*p* < 0.001, Fig. 3d), and impaired endothelial function through heightening ET-1 (*p* < 0.001, Fig. 3e) and vWF (*p* < 0.001, Fig. 3f) levels. MiR-146b-5p knockdown demonstrated alleviative effects on ox-LDL-stimulated HCAEC injury by attenuating these ox-LDL-induced cell impairment through suppressing inflammatory cytokine production (IL-1β: *p* = 0.003; TNF-α: *p* < 0.001), restoring redox balance (MDA: *p* < 0.001; SOD: *p* < 0.001), and improving endothelial function (ET-1: *p* < 0.001; vWF: *p* < 0.001).

### Downstream targets of miR-146b-5p and the GO and KEGG analysis

**Fig. 4:**
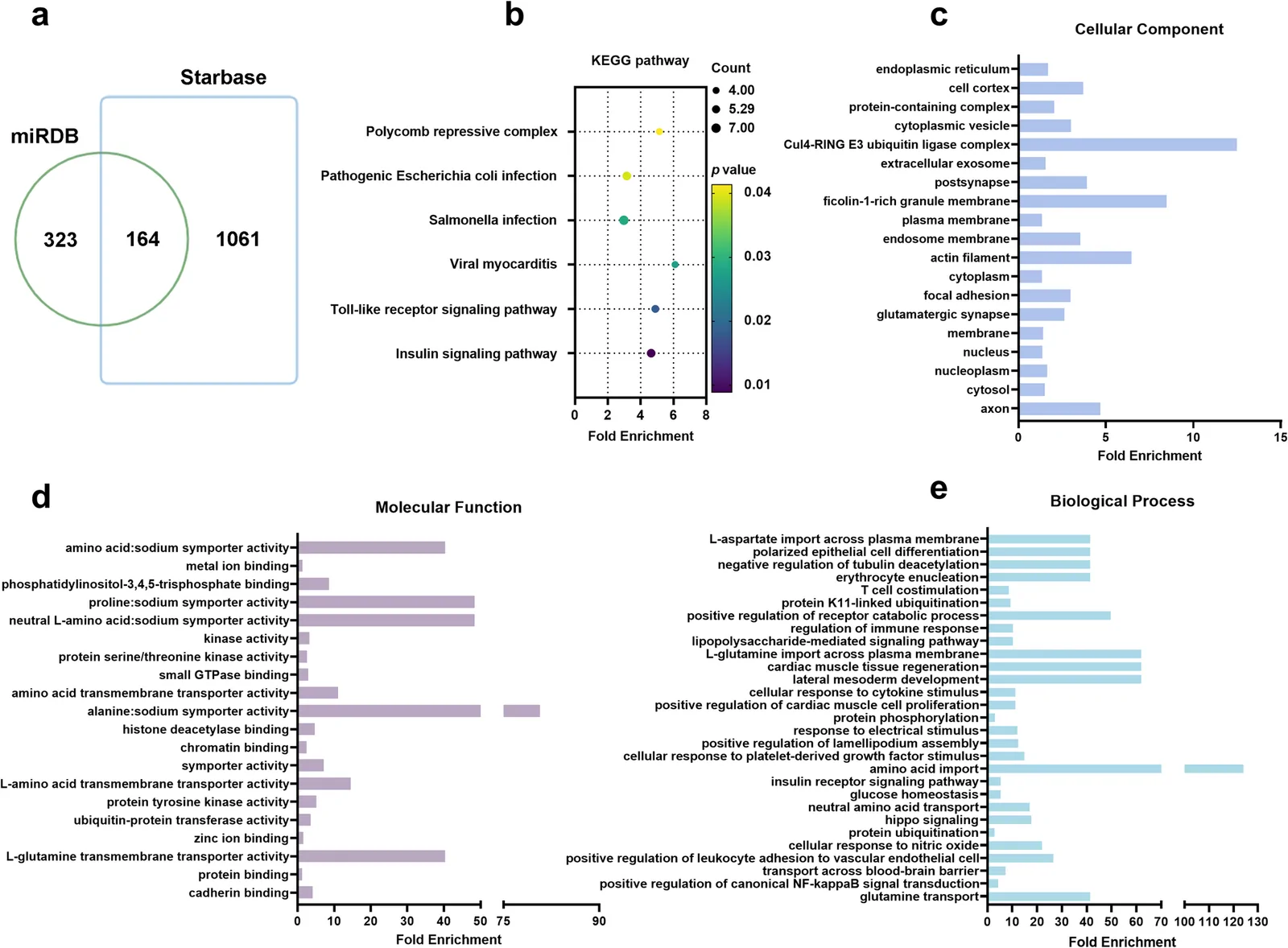
Bioinformatics analysis on miR-146b-5p downstream targets. **a** Venn diagram of predicted target genes from miRDB and Starbase, with 164 shared genes. **b** KEGG pathway enrichment analysis. **c**-**e** GO enrichment analysis for cellular component (**c**), molecular function (**d**), and biological process (**e**)

The comprehensive bioinformatics analysis identified 164 overlapping downstream target genes of miR-146b-5p through integration of Starbase and miRDB databases (Fig. 4a). Functional annotation revealed these targets participate in three major biological pathways including Salmonella infections, Toll-like receptor pathway, and insulin signaling pathway as shown by KEGG analysis (Fig. 4b). GO classification further demonstrated their involvement in CC (cytosol, nucleus, cytoplasm, Fig. 4c), MF (protein binding, metal ion binding, cadherin binding, Fig. 4d), and BP (protein ubiquitination, positive regulation of NF-κB signaling, protein phosphorylation, Fig. 4e).

### Interaction relationship between miR-146b-5p and PRKAR2A in AMI

**Fig. 5:**
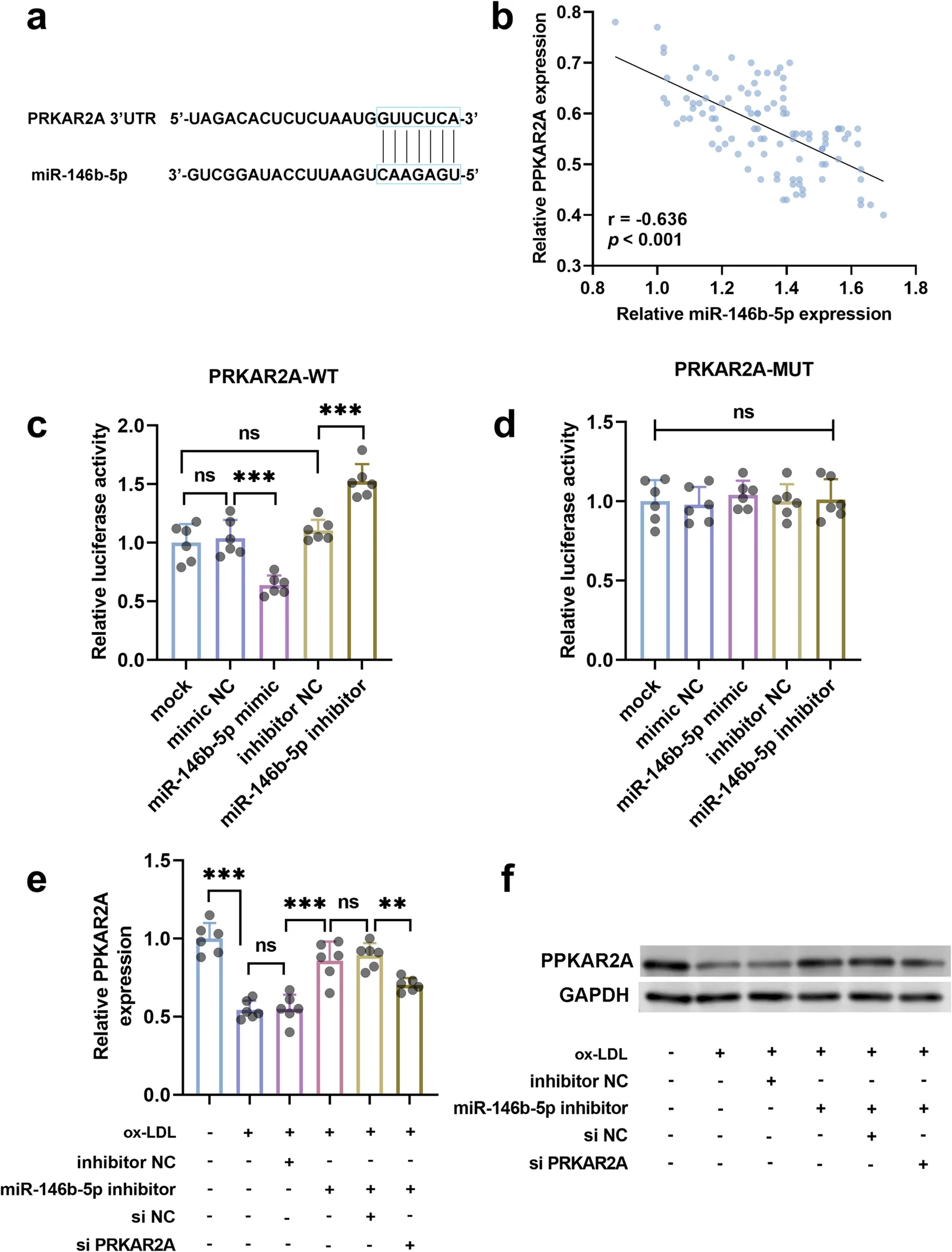
Interaction between miR-146b-5p and PRKAR2A. **a** Binding sites of miR-146b-5p on PRKAR2A; (**b**) Negative correlation between miR-146b-5p and PRKAR2A expression in AMI; (**c**-**d**) Dual-luciferase reporter assays confirmed direct regulatory interaction between miR-146b-5p and PRKAR2A; (**e**) Downregulation of PRKAR2A in ox-LDL-induced HCAECs was upregulated by miR-146b-5p knockdown, which was further attenuated by PRKAR2A suppression; (**f**) Protein levels of PRKAR2A in ox-LDL-induced HCAECs was downregulated and could be upregulated by miR-146b-5p knockdown, which was further reversed by PRKAR2A silencing. ns: no significance, **p < 0.01, ***p < 0.001

The molecular interaction between miR-146b-5p and its target gene PRKAR2A was systematically characterized through multi-level experimental validation. Bioinformatics prediction (miRDB) identified specific binding sites for miR-146b-5p on PRKAR2A (Fig. 5a), which was further clinically supported by the inverse correlation between serum miR-146b-5p levels and PRKAR2A expression in AMI subjects (*r* = -0.636, *p* < 0.001, Fig. 5b). Functional validation through dual-luciferase reporter assays confirmed the direct regulatory role of miR-146b-5p on PRKAR2A, demonstrating miR-146b-5p overexpression suppressed PRKAR2A luciferase activity (*p* < 0.001) that could be rescued by miR-146b-5p inhibition (*p* < 0.001, Fig. 5c), while mutation of the binding site abolished this effect (*p* > 0.05, Fig. 5d). In addition, ox-LDL-induced downregulation of PRKAR2A in HCAECs (*p* < 0.001) was partially reversed by miR-146b-5p suppression (*p* < 0.001), an effect that was subsequently attenuated upon PRKAR2A knockdown (*p* = 0.008, Fig. 5e). Western blot analysis showed the same expression trend of PPKAR2A in HCAECs as downregulated PPKAR2A expression was upregulated by the miR-146b-5p inhibition and could be further suppressed by PPKAR2A silencing (Fig. 5f).

### PRKAR2A suppression reversed the protective effect of miR-146b-5p knockdown in ox-LDL-induced HCAEC injury

**Fig. 6:**
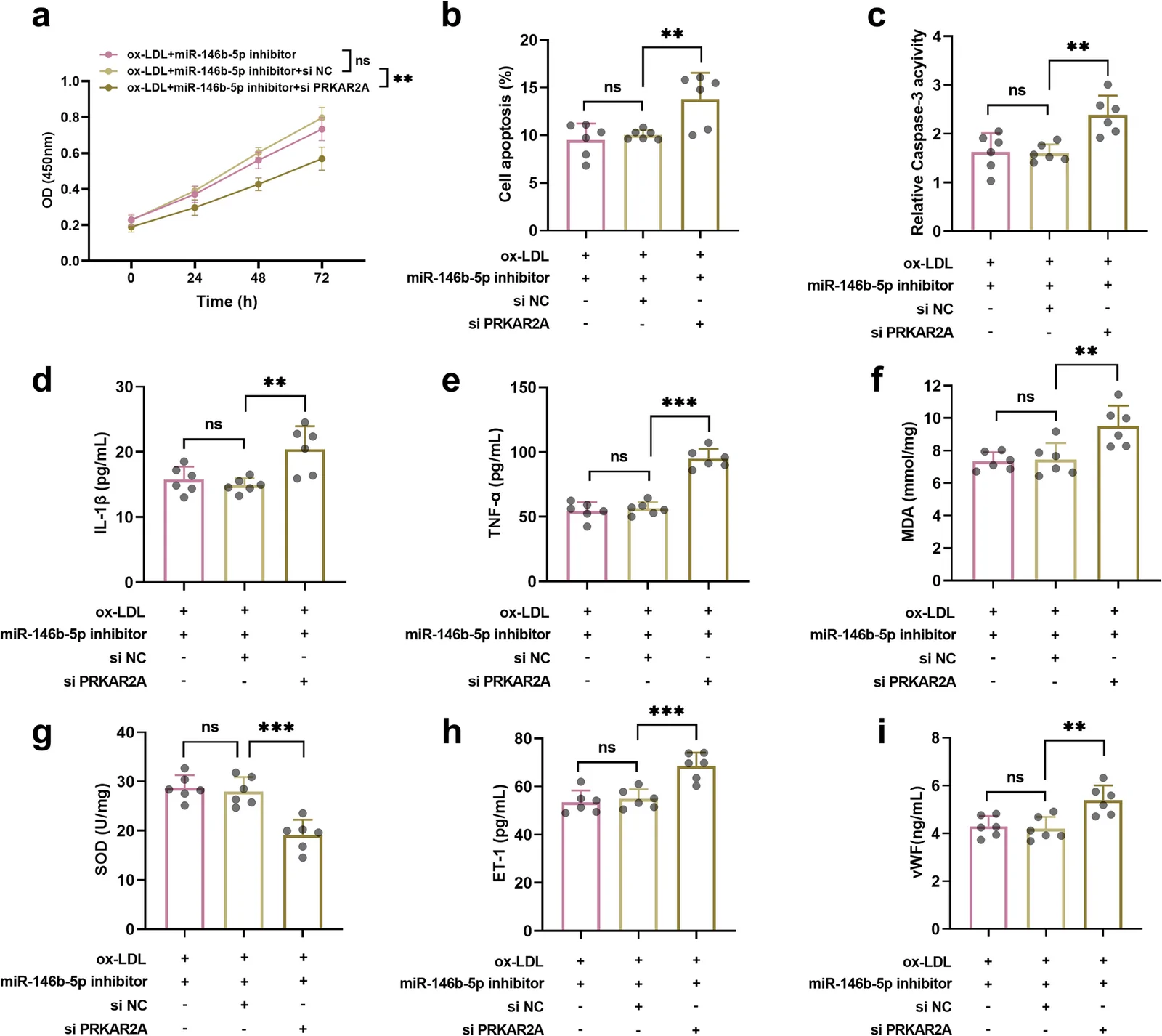
PRKAR2A suppression reversed the protective effect of miR-146b-5p suppression in ox-LDL-induced HCAEC injury. **a**-**i** PRKAR2A knockdown counteracted the beneficial effects of miR-146b-5p suppression by inhibiting cell proliferation (**a**), promoting apoptosis (**b**), and (**c**) enhancing caspase-3 activity (**c**), while also exacerbating inflammation through increased IL-1β (**d**) and TNF-α (**e**) levels, intensifying oxidative stress via elevated MDA levels (**f**) and reduced SOD activity (**g**), and suppressing endothelial dysfunction markers as shown by decreased levels of ET-1 (**h**) and vWF (**i**). ns: no significance, **p < 0.01, ***p < 0.001

PRKAR2A knockdown abrogated the cytoprotective effects of miR-146b-5p inhibition in ox-LDL-induced HCAECs, as demonstrated by impaired proliferative capacity (*p* = 0.010, Fig. 6a), increased apoptotic cell death (*p* = 0.010, Fig. 6b) accompanied by elevated caspase-3 activity (*p* = 0.003, Fig. 6c). PRKAR2A suppression also attenuated multiple beneficial effects originally conferred by miR-146b-5p suppression in ox-LDL-stimulated HCAECs by restoring pro-inflammatory cytokine levels (IL-1β: *p* = 0.004, Fig. 6d; TNF-α: *p* < 0.001, Fig. 6e), re-establishing oxidative stress response (MDA: *p* = 0.007, Fig. 6f; SOD: *p* < 0.001, Fig. 6g), and re-exacerbating endothelial dysfunction parameters (ET-1: *p* = 0.001, Fig. 6h; vWF: *p* = 0.003, Fig. 6i).

## Discussion

MiRNAs are stable, tissue-specific non-coding RNAs with high diagnostic potential due to their disease-responsive expression patterns in biofluids [24]. MiR-146b-5p, in particular, plays critical roles in tumorigenesis [25, 26], pulmonary disorders [27], reproductive complications [28], neurological conditions [29], and wound repair [30]. Despite its established involvement in these disease contexts, the functional significance of miR-146b-5p in AMI remains unexplored. In the present study, we systematically characterized the expression profile, clinical function, and molecular regulatory mechanisms of miR-146b-5p in the progression of AMI, providing novel insights into AMI clinical treatment.

This study demonstrated that miR-146b-5p was significantly upregulated in AMI patients, establishing its potential as a diagnostic biomarker as validated by ROC curve analysis. The pathophysiology of AMI is intricately linked to dyslipidemia, where elevated TC and LDL-C, combined with reduced HDL-C, collectively drive atherosclerotic plaque progression and coronary thrombosis [31, 32], observations that were replicated in our AMI patient cohort. Following myocardial injury, the release of cardiac-specific biomarkers, cTnI and CK-MB, into circulation serves as a direct indicator of myocardial damage, while elevated hs-CRP levels reflect systemic inflammation triggered by plaque destabilization and cardiomyocyte necrosis [33–35]. Concurrently, the loss of contractile function in necrotic myocardial tissue compromises LVEF, a critical parameter of cardiac dysfunction observed in AMI patients [36]. Notably, serum miR-146b-5p levels exhibited robust correlations with hs-CRP, CK-MB, cTnI, and LVEF, suggesting its mechanistic involvement in AMI progression. Clinically, miR-146b-5p expression escalated with Killip classification grades, a quantitative measure of heart failure severity [37], thereby associating miR-146b-5p with AMI acuity. The independent prognostic value of miR-146b-5p for adverse outcomes in AMI was further confirmed through the comprehensive K-M and Cox regression analyses. These multifaceted findings collectively position miR-146b-5p as a dual-functional biomarker with both diagnostic and prognostic capabilities, offering clinicians a novel tool for risk stratification and therapeutic monitoring. It also must be noted that although hyperglycemia and hyperlipidemia are well-established risk factors for AMI [38, 39], and would be expected to be more prevalent in the AMI group, an attempt was made to select a study population with balanced baseline characteristics between the AMI and healthy control groups to minimize potential confounding effects on miR-146b-5p expression. Indeed, neither hyperglycemia nor hyperlipidemia showed statistically significant differences between the two groups. The lack of statistical significance, despite the known pathophysiological association, likely reflected the effort to achieve comparability at baseline. Therefore, the observed differences in miR-146b-5p levels between the two groups were unlikely to be substantially confounded by these metabolic factors, and the conclusions remained valid.

To elucidate the role of miR-146b-5p in AMI-induced endothelial injury, this study employed ox-LDL-induced HCAECs as an in vitro model. Coronary endothelial injury caused by ox-LDL is a primary cause of AMI [40]. Ox-LDL induces endothelial dysfunction, inflammation, and oxidative stress, leading to a prothrombotic state (via elevated vWF), vasoconstriction, and hypoperfusion (via increased ET‑1) [41–43]. These mechanisms collectively trigger myocardial ischemia and infarction. In this study, ox-LDL-induced HCAEC damage manifested as impaired proliferation, elevated apoptosis, increased inflammatory cytokines (IL-1β, TNF-α), and oxidative stress (higher MDA levels and reduced SOD activity), all of which recapitulate the ischemic AMI microenvironment and exacerbate myocardial injury. Ox-LDL exacerbates endothelial dysfunction through the synergistic regulation of ET-1 (a potent vasoconstrictor) and vWF (a platelet-adhesion glycoprotein) [44, 45]. This interplay between ET-1 and vWF forms a vicious cycle: ET-1 stimulates vWF release, promoting platelet aggregation, while vWF-mediated thrombosis further amplifies the vasoconstrictive effects of ET-1 [46, 47]. This positive feedback loop might potentially accelerate AMI progression, thereby perpetuating the pathological cycle. Notably, miR-146b-5p expression was significantly upregulated in ox-LDL-injured HCAECs, and its suppression markedly improved cell viability, reduced apoptosis, alleviated inflammation/oxidative stress, and attenuated endothelial dysfunction (as evidenced by reduced ET-1/vWF levels). These findings collectively suggested that upregulated miR-146b-5p played a pathogenic role in endothelial injury, positioning it as a promising therapeutic target for AMI-related endothelial dysfunction.

Bioinformatics analysis identified PRKAR2A as a downstream target of miR-146b-5p, with its interaction confirmed by dual-luciferase reporter assays and a negative correlation between serum PRKAR2A expression and miR-146b-5p levels in AMI patients. Ox-LDL-induced HCAECs showed downregulated PRKAR2A expression, which was reversed by miR-146b-5p suppression, further validating their regulatory relationship. Functional annotation via GO and KEGG analyses confirmed the involvement of PRKAR2A in critical biological processes (e.g., cytosol localization, protein binding) and signaling pathways (e.g., insulin signaling). The insulin signaling pathway has also been reported to be associated with myocardial infarction [48]. PRKAR2A has been linked to ischemic disorders, with studies showing its promotion of cerebral ischemia progression (a pathomechanism analogous to AMI) and its association with heart failure [49, 50]. PRKAR2A has also been previously reported to be associated with adiponectin, which is lower in AMI patients [51, 52]. Ox-LDL-injured HCAECs revealed that PRKAR2A knockdown abrogated the protective effects of miR-146b-5p inhibition, as evidenced by exacerbated inflammation, oxidative stress, and elevated endothelial dysfunction markers. These results collectively demonstrated that miR-146b-5p mediated ox-LDL-induced endothelial injury through PRKAR2A regulation, establishing the miR-146b-5p/PRKAR2A axis as a promising target for AMI treatment.

The study findings should be interpreted considering several limitations. The moderate sample size of this study may constrain the generalizability of clinical observations and may not have provided sufficient power to generate narrow confidence intervals, particularly for effect estimates with relatively wide ranges. Therefore, the precision of these hazard ratios should be interpreted with caution, and future studies with larger cohorts are needed to validate our findings. In addition, the regulatory mechanisms of PRKAR2A on downstream pathways remain incompletely characterized. Further animal experiments could enhance mechanistic understanding by validating these findings in vivo. In addition to endothelial cells, vascular smooth muscle cells (VSMCs) also contribute critically to AMI pathophysiology. VSMCs are key components of the atherosclerotic plaque as their proliferation, migration, and phenotypic switching from a contractile to a synthetic phenotype promote plaque formation and instability, ultimately leading to plaque rupture and coronary thrombosis [53]. Moreover, after myocardial infarction, VSMCs participate in adverse ventricular remodeling and fibrosis [53]. Although our study focused on HCAECs because endothelial injury is the initial trigger of atherosclerosis and AMI, future investigations should also examine the effect of miR‑146b‑5p on VSMC function, as cross‑talk between endothelial cells and VSMCs may further modulate disease progression. Furthermore, although ox‑LDL‑induced HCAEC injury is a well‑established in vitro model that recapitulates key features of endothelial dysfunction in AMI, it was acknowledged that using only one type of endothelial injury model may not fully represent the complexity of endothelial damage in patients. Additional models (e.g., hypoxia/reoxygenation, inflammation‑triggered injury, or shear stress‑induced damage) could provide complementary insights into the role of miR‑146b‑5p under different pathological stimuli. Future research directions should include: (1) expanding cohort sizes from a multi-center to improve statistical power, (2) employing multi-omics approaches for comprehensive pathway analysis, (3) utilizing clinically relevant animal models that better recapitulate human disease pathophysiology, and (4) incorporating additional endothelial injury models to validate the robustness of our findings. Addressing these limitations through these methodological advancements would strengthen the translational potential of the research.

## Conclusions

MiR-146b-5p was upregulated in AMI patients and correlated with AMI severity. In addition, miR-146b-5p upregulation could serve as a potential diagnostic biomarker and prognostic indicator for AMI. In mechanism, miR-146b-5p inhibition mitigated ox-LDL-induced endothelial injury by reducing apoptosis, inflammation, and promoting proliferation through regulating the PRKAR2A expression. These findings highlighted miR-146b-5p as a potential biomarker and therapeutic target for AMI, and might provide novel insights for AMI clinical management.

## Supplementary Information

Supplementary Material 1: Fig S1. Study workflow.

Supplementary Material 2: Fig S2. Western blot analysis of PRKAR2A.

## Data Availability

All data generated or analyzed during this study are included in this article. Further enquiries can be directed to the corresponding author.
